# Regulation of nuclear factor erythroid-2-related factor 2 as a potential therapeutic target in intracerebral hemorrhage

**DOI:** 10.3389/fnmol.2022.995518

**Published:** 2022-09-29

**Authors:** Yuan Zhang, Wanpeng Yu, Yingying Liu, Wenguang Chang, Man Wang, Lei Zhang

**Affiliations:** ^1^Institute of Translational Medicine, The Affiliated Hospital of Qingdao University, Qingdao University, Qingdao, China; ^2^Medical College, Qingdao University, Qingdao, China; ^3^Institute of Translational Medicine, The Affiliated Hospital of Hangzhou Normal University, Hangzhou, China

**Keywords:** intracerebral hemorrhage, Nrf2, oxidative stress, therapeutic targets, activators

## Abstract

Hemorrhagic stroke can be categorized into several subtypes. The most common is intracerebral hemorrhage (ICH), which exhibits significant morbidity and mortality, affecting the lives of millions of people worldwide every year. Brain injury after ICH includes the primary injury that results from direct compression as well as stimulation by the hematoma and secondary brain injury (SBI) that is due to ischemia and hypoxia in the penumbra around the hematoma. A number of recent studies have analyzed the mechanisms producing the oxidative stress and inflammation that develop following hematoma formation and are associated with the ICH induced by the SBI as well as the resulting neurological dysfunction. Nuclear factor erythroid-2-related factor 2 (Nrf2) is a critical component in mediating oxidative stress and anti-inflammatory response. We summarize the pathological mechanisms of ICH focusing on oxidative stress and the regulatory role of Nrf2, and review the mechanisms regulating Nrf2 at the transcriptional and post-transcriptional levels by influencing gene expression levels, protein stability, subcellular localization, and synergistic effects with other transcription factors. We further reviewing the efficacy of several Nrf2 activators in the treatment of ICH in experimental ICH models. Activation of Nrf2 might produce antioxidant, anti-inflammatory, and neuron-protection effects, which could potentially be a focus for developing future treatments and prevention of ICH.

## Introduction

The most common subtype of hemorrhagic stroke is intracerebral hemorrhage (ICH), which comprises about 10–15% of all cases of stroke. ICH exhibits significant morbidity and mortality, affecting the lives of millions of people worldwide every year (Al-Kawaz et al., 2020). Brain injury following ICH includes primary brain injury that is due to direct compression and stimulation by the hematoma, as well as secondary brain injury (SBI) that is due to the ischemia and hypoxia occurring in the penumbra around the hematoma (Babu et al., 2012). In the first few hours after the onset of ICH, extravasated blood and the hematoma cause the primary brain injury due to the pressure on and ultimate destruction of the adjacent tissues. The primary brain injury also causes inflammation in the surrounding tissue, activation of thrombin, and lysis of erythrocytes, all of which induce brain edema as well as long-lasting injuries. Furthermore, the components and metabolic products of late-stage hematomas induce direct toxicity and inflammation leading to SBI (Bian et al., 2020). However, the SBI might produce significant neurological deficits and could even cause the death of the patient (Liu et al., 2020). Numerous recent studies have examined the mechanisms associated with the inflammation and oxidative stress that occur following hematoma formation and are involved in the SBI produced by ICH and the resulting neurological dysfunction (Wang Z. et al., 2018).

Nuclear factor erythroid-2-related factor 2 (Nrf2) has a critical role in regulating oxidative stress and in ameliorating brain damage, which may be a target of interest after ICH (Bellezza et al., 2018). Nrf2 controls the expression of heme oxygenase-1 (HO-1) and multiple other proteins with antioxidant and anti-inflammatory effects, and plays an important role in reducing brain damage after ICH (Deng et al., 2021; Shen et al., 2021). Studies show that Nrf2 nuclear accumulation can reduce brain injury in the early stage of ICH (Wang et al., 2007). After ICH, Nrf2 is translocated into the nucleus, where it regulates the expression of many antioxidant enzymes by combining with an antioxidant response element (ARE) (Cheng et al., 2020). These changes in gene expression carry out essential roles in improving SBI. Thus, regulation of Nrf2 can be used as a potential therapeutic target in ICH.

Here, we summarize the pathological mechanisms of ICH focusing on oxidative stress and the regulatory role of Nrf2, further reviewing the efficacy of several Nrf2 activators in the treatment of ICH in experimental ICH models.

## The role of oxidative stress in the pathophysiology of intracerebral hemorrhage

The poor prognosis of ICH is due to the SBI that follows the primary brain injury. The toxic effects of hemoglobin, iron, and other blood components on the brain tissue are thought to be a driver of SBI (Wei et al., 2020). Starting several hours after ICH onset, the primary injury activates thrombin and causes lysis of erythrocytes, which induces neuroinflammation and oxidative stress. These events promote brain edema to occur, resulting in serious, long-lasting injury to the brain (Zheng et al., 2016; Xue and Yong, 2020). Current research shows that the inflammatory response occurring after ICH is highly associated with oxidative stress, resulting in brain damage. The oxidative stress also leads to inflammation, which aggravates the damage to the neurovascular system and leads to neurological deficits.

In the early stage of ICH, the erythrocyte lysate activates microglia and causes inflammatory cells, including leukocytes and macrophages, to infiltrate the affected tissues, resulting in the release and accumulation of pro-inflammatory mediators and ultimately neuroinflammation. In addition, neutrophil activation also damages the respiratory chain, causing a release of large amounts of reactive oxygen species (ROS), which leads to the occurrence of oxidative stress (Neulen et al., 2019).

Oxidative stress is considered to be an essential component in the occurrence of SBI after ICH. It is involved in many critical stages of the physiological and pathological responses that take place during ICH. Iron ions, heme, activation of thrombin, and lysis of red blood cells (RBCs) following ICH initiate reactive free radical production, including reactive nitrogen species (RNS) and ROS, which are the primary causes of oxidative stress (Hu et al., 2016). Studies have shown that the products of RBC lysis, including iron ions and heme generate large amounts of ROS, which directly lead to neuronal damage. The inflammation that results from ICH also generates considerable amounts of ROS that can precipitate neuronal cell death (Huang et al., 2002). In addition, the presence of excessive ROS will directly cause the production of acute pro-inflammatory cytokines, including tumor necrosis factor α (TNF-α) and interleukin-10 (IL-10). ROS also can cause the activation of nuclear factor-κB (NF-κB), which has a critical role in promoting the inflammatory reaction (Ma et al., 2014; Chen et al., 2022). During ICH-induced inflammation, the neutrophil activation causes damage to the respiratory chain, releasing excessive amounts of ROS and nitric oxide. Consequently, a considerable amount of superoxide dismutase (SOD) is consumed to eliminate the free radicals, eventually leading to lipid peroxidation. When excessive lipid peroxidation occurs, the physical properties of the cell membranes are altered, which can result in covalent protein and nucleic acid modifications, producing neuronal damage (Gaschler and Stockwell, 2017).

Even though excessive oxidative stress is known to be detrimental, normal levels of activation of oxidative stress are necessary to maintain the normal physiological processes in the body. Oxidative stress also can serve as a defensive mechanism to kill pathogens through modulation of the immune system. It has been reported that limited activation of oxidative stress could play a significant role in the treatment of ICH and prevention of ICH-induced SBI.

## Regulation of nuclear factor erythroid-2-related factor 2 pathway as a potential therapeutic target in intracerebral hemorrhage

### The role of nuclear factor erythroid-2-related factor 2/antioxidant response element pathway in intracerebral hemorrhage

Nuclear factor erythroid-2-related factor 2 has a critical role in regulating the xenobiotic-activated receptor (XAR) that exhibits an essential role in regulating oxidative stress and reducing oxidative damage after ICH (Bellezza et al., 2018). Keap1 is the master intracellular regulator of Nrf2 (Baird and Yamamoto, 2020). Under normal physiological conditions, Nrf2 interacts with Keap1 and is anchored in the cytoplasm (Bellezza et al., 2018; Baird and Yamamoto, 2020). Nrf2 binds to the DGR region of Keap1 *via* the Neh2 domain of Nrf2, forming a homodimer that binds Cullin3 (Cul3), which serves as an adaptor to the Cul3-type E3 ubiquitin ligase complex (Cul3/Rbx1 E3 ubiquitin). This complex then carries out ubiquitination in the cytoplasm. Keap1 serves as a substrate for Cul3/Rbx1 E3 ubiquitin, which can promote the ubiquitination of Nrf2 resulting in its rapid degradation from the proteasome, which serves to maintain Nrf2 levels. During conditions that produce oxidative stress, including excessive production of ROS, Nrf2 and Keap1 disconnect, and Nrf2 translocates into the nucleus. There, Nrf2 is able to dimerize with small members of the Maf family and join with the ARE. Nrf2 can coordinate anti-oxidative stress processes by means of AREs that are located in target gene regulatory regions. An ARE is characterized as a cis-acting DNA enhancer motif that is found in antioxidant gene promoter regions (Buendia et al., 2016). When Nrf2 binds to an ARE, the transcriptional activation of numerous essential endogenous antioxidants is regulated, and harmful molecules, including ROS, are removed. When the Nrf2-ARE signaling pathway is activated, the expression of genes associated with related endogenous defenses against toxic and oxidative insults also are activated in various tissues and cells *in vivo*. The genes that are activated include genes for antioxidant enzymes, various detoxification proteins, and other anti-inflammatory factors. When the expression of these anti-inflammatory and antioxidants is elevated in neurons and glial cells, the oxidative stress caused by RBC lysis following ICH is countered.

Studies show that Nrf2 nuclear accumulation can reduce brain injury in the early stage of ICH. Numerous animal experiments have revealed that knocking out the Nrf2 gene results in more pronounced hematoma volumes, neurological deficits, greater blood–brain barrier (BBB) disruption, and increased brain edema after ICH (Wang et al., 2007; Cheng et al., 2021). Also, following ICH, Nrf2 is translocated into the nucleus, where it regulates the expression of many antioxidant enzymes by combining with an ARE. These changes in gene expression carry out essential roles in improving SBI. Many traditional Chinese medicines such as isoliquiritigenin and luteolin can effectively increase the gene expression of antioxidant enzymes such as catalase (CAT), SOD, and glutathione S-transferase (GST) in brain tissue after ICH by activating the Nrf2 signaling pathway. This activation has been shown to dramatically alleviate neurological deficits and disruption of the BBB, reduce brain edema, and ameliorate neuronal degeneration (Zeng et al., 2017; Tan et al., 2019). On the other hand, when the Nrf2 gene is silenced, brain edema and neuronal degeneration are aggravated, which produces increases in hemorrhage and infiltration of leukocytes, elevated production of ROS, and excessive DNA damage. Thus, the Nrf2-ARE signaling pathway has a substantial role in the anti-oxidative and anti-inflammatory responses, as well as the protection of neurons in ICH and ICH-induced SBI.

### MicroRNAs regulate nuclear factor erythroid-2-related factor 2 expression in intracerebral hemorrhage

MicroRNAs (miRNAs) are short RNAs that consist of lengths in the range of 21–24 base pairs. MiRNAs carry out critical roles in the regulation of gene expression at the post-transcriptional level. They specifically bind to the 3′ untranslated region (3′-UTR) of the target mRNAs causing their degeneration or inhibiting their translation, all of which will result in decreased levels of protein (Liu et al., 2021, 2022).

For example, it has been shown that miR-27b directly targets the 3′-UTR of the Nrf2 mRNA, which was reduced in ICH. When miR-27b levels have decreased, this results in increased Nrf2 expression, as well as increased levels of Hmox1, Sod1, and Nqo1. Furthermore, inhibition of miR-27b has been shown to decrease the oxidative and inflammatory injury associated with ICH, which raises the possibility of using miRNAs in the treatment of ICH (Xu et al., 2017).

MiR-93 is another miRNA that has been reported to function as a therapeutic target for ICH. MiR-93 was markedly downregulated, and the mRNAs of TGF-β1 and Nrf2 were increased after ICH. Increasing the levels of TGF-β1 or decreasing miR-93 expression after ICH could increase the expression of Nrf2. MiR-93 can bind to the 3′-UTR of the Nrf2 mRNA and the 3′-UTR of the TGF-β1 mRNA. TGF-β1 3′-UTR functions as a competing endogenous RNA (ceRNA) by sponging miR-93, which can compete with Nrf2 for binding to miR-93, thereby releasing Nrf2, leaving Nrf2 mRNA free to be translated (Wang et al., 2021).

MiR-183-5p also exerts an anti-inflammatory and antioxidant role in ICH. Wang et al. show that miRNA-183-5p expression is significantly decreased after ICH in mice. MiR-183-5p protects brain tissue and improves neurologic function after ICH by targeting and inhibiting HO-1 expression, and has a negative correlation with Nrf2 phosphorylation, albeit indirectly (Wang et al., 2020).

Brain and muscle Arnt-like protein 1 (BMAL1) is the core element of the circadian clock, which is reported involved in anti-oxidative stress and inflammatory responses. Previous study show that Bmal1 directly regulates the Nrf2 signaling pathway by binding to its response element E-box (Early et al., 2018). Recently, Gong et al. (2021) found that miRNA-155 could negative regulate BMAL1 in mRNA level. Inhibiting miRNA-155 can promote the expression of BMAL1 and further activate the Nrf2 signaling pathway to attenuate brain injury induced by ICH.

### Regulation of nuclear factor erythroid-2-related factor 2 protein stability in intracerebral hemorrhage

#### Small ubiquitin-related modification of nuclear factor erythroid-2-related factor 2

Small ubiquitin-related modifier (SUMO) proteins can covalently modify lysine residues of some proteins, which is known as SUMOylation. SUMOylation primarily affects protein stability and protein-protein interactions, and also is reported to modulate nuclear and intranuclear localization of some proteins and antagonize ubiquitylation, thereby exerting many critical physiological functions. The human genome encodes three different functional isoforms of SUMO, including SUMO-1, SUMO-2, and SUMO-3. Studies have shown that Nrf2 is ubiquitinated by Arkadia/RNF111, a poly-SUMO-specific E3 ubiquitin ligase, which stabilizes Nrf2 (McIntosh et al., 2018). The lysine residue 110 (K^110^) located in the ^109^PKSD^112^ motif (in the Neh4 domain) of the human Nrf2 is a potential SUMO-target, serving as the SUMO-2 binding site. SUMOylation of the Nrf2 K^110^ site by SUMO-2 promotes the stability and translocation of human Nrf2 to the nucleus and transactivation of antioxidant genes, such as HO-1 (Walters et al., 2021). Therefore, SUMOylation of Nrf2 by SUMO-2 maintains Nrf2 levels and promotes the nuclear translocation and transcriptional activity of Nrf2, which might be useful as a novel therapy for ICH.

#### p62-dependent autophagy prolongs nuclear factor erythroid-2-related factor 2 activity

Prolonged Nrf2 activity might depend on p62-mediated autophagy. p62 is a type of autophagy-adaptor protein that binds with ubiquitylated protein aggregates and delivers them to the autophagosomes (Jiang et al., 2015). Therefore, p62 degrades Keap1 through autophagy by binding with Keap1. Studies have shown that p62 is able to interact with the Nrf2 binding site, which is found on the Keap1 protein. This interaction allows simultaneous competition with the Nrf2 protein. In addition, p62 can sequester Keap1 in the autophagosome for degradation that occurs during autophagy, which ultimately prevents Nrf2 protein degradation, prolonging Nrf2 activity. It has been reported that the p62/Keap1/Nrf2 pathway plays a central role in the pathological mechanism underlying cerebral hemorrhage. For example, luteolin enhances autophagy and Nrf2 transfer into the nucleus by activating the p62/Keap1/Nrf2 pathway, which attenuates the ICH-induced SBI observed in ICH patients (Tan et al., 2019). Luteolin treatment decreased p62 protein levels and increased LC3II protein levels, which promoted the activation of autophagy. Through this process, the Keap1 protein was degraded through the autophagy pathway, and the level of the Keap1 protein was decreased. Reducing ubiquitination of Nrf2 by disengaging it from Keap1 maintains Nrf2 protein levels and promotes its movement into the nucleus, further increasing the downstream antioxidant protein expression levels, including HO-1 and reduced nicotinamide adenine dinucleotide phosphate (NADPH): quinine oxidoreductase 1 (NQO1). These processes provide neuroprotection from ICH-induced SBI. Regulation of autophagy was shown to facilitate prolongation of Nrf2 activation in a p62-dependent manner.

### Regulation of nuclear factor erythroid-2-related factor 2 subcellular localization by phosphorylation

Phosphorylation is an essential post-translational modification of Nrf2. It is involved in numerous aspects of the regulation of Nrf2. Furthermore, Nrf2 provides several potential sites for phosphorylation for a range of protein kinases. These modifications in Nrf2 phosphorylation might influence the degradation of the Nrf2 proteasome, translocation of Nrf2 into the nucleus, binding of Nrf2 to the ARE sequence, and export of Nrf2 from the nucleus.

#### Phosphatidylinositol-4,5-bisphosphate 3-kinase/protein kinase B/nuclear factor erythroid-2-related factor 2 pathway

The phosphatidylinositol-4,5-bisphosphate 3-kinase (PI3K)/protein kinase B (Akt) pathway is an important intracellular signaling pathway that is involved in multiple cellular processes. PI3K has been shown to be an intracellular phosphatidylinositol kinase that exhibits serine/threonine (Ser/Thr) kinase activity as well as phosphatidylinositol kinase activity, which activates Akt through the phosphorylation of Akt at the Ser^473^ residue. Recently evidence has demonstrated that the PI3K/Akt pathway is associated with Nrf2 phosphorylation at ser^40^, and inhibition of PI3K or Akt will diminish the activation of Nrf2, which is critically involved in a number of oxidative stress-related diseases (Liao et al., 2020). Thus, the PI3K/Akt/Nrf2 pathway might be a likely target for preventing oxidative stress-induced SBI in ICH patients.

For example, NDP-MSH significantly reduced ROS and attenuated brain edema induced by ICH through activation of the PI3K/Akt/Nrf2 pathway. Treatment with NDP-MSH increased p-PI3K, p-Akt, p-Nrf2, and Bcl-2 expression in brain tissue located in the perihematomal area 24 h after ICH onset. These changes were accompanied by decreased levels of cleaved caspase-3. When the PI3K inhibitor LY294002 was utilized to block PI3K/Akt signaling, the antiapoptotic and antioxidant influence of NDP-MSH was lost. This loss was caused by the reversal of expression changes that occurred in p-Nrf2, Bcl-2, and caspase-3. These results indicate that NDP-MSH promotes the phosphorylation of Nrf2 through PI3K/Akt signaling, which increases Nrf2 activity and triggers antioxidant gene expression (Fu S. et al., 2020).

#### Glycogen synthase kinase 3β/nuclear factor erythroid-2-related factor 2 pathway

Glycogen synthase kinase 3β (GSK-3β) has been demonstrated to be a serine/threonine protein kinase. GSK-3β functions as an inhibitor, which is activated by phosphorylation of the tyrosine 216 residue or de-phosphorylation at the serine 9 residue, which both directly or indirectly inactivate Nrf2. GSK-3β phosphorylates Nrf2 at Ser^334–338^ and negatively regulates the activation of Nrf2 by exporting it from the nucleus, causing its degradation (Bian et al., 2021).

Glycogen synthase kinase 3β indirectly modulates Nrf2 *via* Fyn phosphorylation (Jain and Jaiswal, 2007). Fyn-mediated phosphorylation of Nrf2 occurs at tyrosine 568 inside the nucleus. The phosphorylated Nrf2 Y568 binds to Crm1 leading to Nrf2 export out of the nucleus and its subsequent degradation (Jain and Jaiswal, 2007). However, p-Akt can phosphorylate GSK-3β at Ser9 and negatively regulate GSK-3β, thereby suppressing GSK-3β from phosphorylation Nrf2 and promoting the activation of Nrf2 by its translocation into the nucleus (Bian et al., 2021).

For example, Apelin 13 protected against ischemic stroke-induced inflammation and oxidative stress through activating GSK-3β/Nrf2 pathway. Apelin 13 treatments induced phosphorylation of GSK-3β at serine 9, further promoted the nuclear translocation of Nrf2 and the expression of downstream antioxidant proteins (Duan et al., 2019). Rana et al. (2022) demonstrated that the expression of p-Gsk-3β (Ser9) and Nrf2 protein were significant decrease in zebrafish model of ICH. Rutin treatment reduced the hematoma size and ROS production trough upregulation of p-Gsk-3β (Ser9) and Nrf2 protein in the zebrafish brains. These suggest that negative regulation of GSK-3β activity through phosphorylation of serine 9 could promote Nrf2 activation and overcome oxidative stress and inflammatory responses.

#### Protein kinase C α/nuclear factor erythroid-2-related factor 2 pathway

Protein kinase C (PKC) is reported to phosphorylate Nrf2 at Ser^40^, which is in the Neh2 domain. This results in Nrf2 disengaging from Keap1. Then, Nrf2 is translocated into the nucleus, where Nrf2 activity is promoted for downstream antioxidant genes. For example, melatonin protects astrocytes from the toxic effects of hemin by activating the PKCα/Nrf2 signaling pathway (Chen et al., 2019). Administration of melatonin induces the phosphorylation of PKCα (p-PKCα), upregulation of Nrf2, and translocation of Nrf2 in astrocytes. These changes result in the upregulation of HO-1, which is, at least in part, responsible for a reduction in the accumulation of ROS and cell apoptosis induced by hemin. When treated with a PKC inhibitor, the upregulation of Nrf2 and HO-1 protein expression caused by melatonin administration was reversed. Therefore, the PKCα/Nrf2 signaling pathway activation could be a beneficial process to provide neuroprotection after ICH.

#### AMP-activated protein kinase/nuclear factor erythroid-2-related factor 2 pathway

AMP-activated protein kinase (AMPK) is known to accomplish critical regulatory activities in cellular metabolism and energy homeostasis that are involved in cell survival under stress. AMPK phosphorylates Nrf2 at Ser^558^ (or Ser^550^ in mice), which is located in the Neh1 domain, promoting Nrf2 nuclear accumulation. For example, chemerin is able to promote AMPK phosphorylation, which results in increased expression of Nrf2, suppressing neuroinflammation and improving neurological recovery after hemorrhage in the germinal matrix in neonatal rats (Zhang et al., 2018). Phosphorylation of AMPK by activation of AdipoR1 can promote increased Nrf2 expression, which reduced neuroinflammation and oxidative stress injury in ICH mice (Zhao et al., 2021). It is possible that Nrf2 is activated by p-AMPK, and the activated Nrf2 is translocated into the nucleus, where it accumulates. In addition to direct phosphorylation of Nrf2, AMPK can indirectly promote the activity of Nrf2 by phosphorylation of GSK3β. AMPK also can cause the inhibition of GSK-3β activity by phosphorylating Ser^9^, which suppresses the retention of Nrf2 in the cytoplasm by GSK-3β or increases the export of Nrf2 (Park et al., 2018; Duan et al., 2019).

#### Extracellular signal-regulated kinases/nuclear factor erythroid-2-related factor 2 pathway

Extracellular signal-regulated kinases (ERK) 1/2 are a subfamily of MAPKs, and they phosphorylate Ser or Thr residues in downstream proteins, which has a protective effect on oxidative stress and ER stress (Rai et al., 2019). Recent evidence demonstrated that phosphorylated ERK1/2 activates Nrf2 by phosphorylating Nrf2. Therefore, promoting ERK1/2 phosphorylation can increase Nrf2 expression as well as its downstream antioxidant genes, such as HO-1. For example, the ability of albumin to reduce oxidative stress and neuronal apoptosis in ICH rats is accomplished by activating the ERK/Nrf2/HO-1 pathway (Deng et al., 2021). Albumin treatment increased ERK1/2 phosphorylation and Nrf2 and HO-1 expression after ICH in rats. However, the ERK inhibitor U-0126 reversed the albumin-induced ERK1/2 phosphorylation and the increased Nrf2 and HO-1 expression. This indicates that the mechanism of albumin’s protective effect on ICH is through the activation of Nrf2 after phosphorylation of ERK1/2. The mechanism by which ERK1/2 can promote the expression of Nrf2 might be similar to that of other phosphokinases such as AMPK. That is, phosphorylated Nrf2 can translocate into the nucleus to accumulate there to maintain its levels. Similarly, the recombinant CCL17 was reported to have a protective effect in ICH by activating the ERK/Nrf2 pathway. Recombinant CCL17 can promote p-ERK1/2 and Nrf2 expression after ICH (Deng et al., 2020).

#### Casein kinase 2/nuclear factor erythroid-2-related factor 2 pathway

Casein kinase 2 (CK2) is a multifunctional serine/threonine protein kinase, which participates in multiple developmental and stress-responsive processes. CK2 can directly phosphorylate the TA domains of Nrf2, thereby promoting its nuclear localization and transcriptional activity (Apopa et al., 2008). Another study has shown that several Nrf2 phosphorylation sites that are acted on by CK2 are key controlling factors for Nrf2 activity and degradation. CK2-mediated two-step phosphorylation of Nrf2 yields a distinct molecular mass (Mr) of Nrf2 of 98 kDa (Nrf2-98) and 118 kDa (Nrf2-118). Nrf2-98 has been shown to exhibit transcriptional activity (Liu et al., 2020). On the other hand, Nrf2-118 has been shown to have a greater susceptibility to degradation (Wang et al., 2007). The phosphorylated Nrf2 with a Mr of 118 kDa is phosphorylated at Ser and/or Thr sites, and the p-Nrf2 forms are usually detected as both molecular weights. Nevertheless, most drugs exert neuroprotection through CK2 phosphorylation of Nrf2 to promote Nrf2 translocation into the nucleus in ICH. For example, exposure to dimethyl fumarate appears to be neuroprotective through CK2 phosphorylation of Nrf2 in mice with ICH (Iniaghe et al., 2015). It also has been shown that dimethyl fumarate treatment increased cytoplasmic CK2 expression, as well as increased the nuclear expression of p-Nrf2, which conferred neuroprotective effects after ICH through the reduction of brain edema and improvement of functional outcomes.

### Synergistic actions stimulate nuclear factor erythroid-2-related factor 2 activity

#### Peroxisome proliferator-activated receptor γ stimulate nuclear factor erythroid-2-related factor 2 activity

Peroxisome proliferator-activated receptor γ (PPARγ) is reported to be a ligand-activated receptor that is a member of the nuclear hormone receptor family. A heterodimer between PPARγ and the retinoid X receptor (RXR) can form that subsequently joins with the peroxisome proliferator response element (PPRE) in the promoter region of the target genes of PPARγ. Furthermore, PPARγ is known to regulate CD36 and CD163 expression, which allows participation in hematoma clearance after ICH (Wang G. et al., 2018). Studies have shown that PPARγ exerts a synergistic effect with Nrf2 to regulate the expression of related genes involved in the antioxidant response (Dovinova et al., 2020). For example, monascin, which participates in the dual activation of PPARγ and Nrf2, has been shown to improve long-term outcomes by facilitating hematoma clearance. The clearance appears to occur by means of the haptoglobin-hemoglobin-CD163 pathway and also by reducing the iron overload and brain atrophy that was produced in experimental ICH (Fu P. et al., 2020). Duan et al. demonstrated that PPARγ activation by the agonist pioglitazone (PDZ) reduced the neuronal loss occurring in ICH through synergistic actions with Nrf2 and facilitated the protection of neuronal function. Activation of PPARγ promotes the transcriptional activation of Nrf2 by acting synergistically with Nrf2 to increase the expression of Gpx4, thereby inhibiting ferroptosis of neurons after ICH, and promotes the recovery of neural function (Duan et al., 2022a). Therefore, activation of PPARγ by agonists to synergistically promote Nrf2 transcriptional activity to inhibit oxidative stress, inflammation, and ferroptosis is a possible new focus for developing novel treatments for ICH and ICH-induced SBI.

#### Brain and muscle Arnt-like protein 1 stimulate nuclear factor erythroid-2-related factor 2 activity

Brain and muscle Arnt-like protein 1 is a transcription factor and a principal driver of the molecular clock in mammals (Gong et al., 2021). BMAL1 forms a heterodimer with CLOCK, which binds to E-box sites that have locations throughout the genome; this binding influences the rhythmic expression of various clock-controlled genes (Early and Curtis, 2016). The function of BMAL1 promote Nrf2 activity has been confirmed to afford protection in ICH. BMAL1 binds to the Nrf2 gene promoter through an E-box element to increase the expression of Nrf2 and promote the activity of the Nrf2/ARE signaling pathway (Early et al., 2018; Sun et al., 2021). One study found that BMAL1 levels were reduced in the brain of ICH patients. Upregulation of BMAL1 can alleviate oxidative stress and inflammation and attenuate ICH-induced brain edema through increasing Nrf2 expression and activating the Nrf2 signaling pathway (Gong et al., 2021).

In recent years, evidence has shown that BMAL1 and Nrf2 cooperatively regulate the transcription of several antioxidant genes. BMAL1 and Nrf2 can bind to the E-box and ARE in antioxidant gene promoter, respectively. The interaction between BMAL1 and the E-box cooperatively promotes the transcriptional activation of Nrf2 in downstream genes, thereby promoting the expression of antioxidant genes. As Chhunchha et al. (2020) reported BMAL1 and Nrf2 cooperatively regulated Prdx6 transcription to protect cells against aging or oxidative stress. Although there is no direct evidence that the synergistic effect of Bmal1 and Nrf2 is involved in the pathological mechanism of ICH, its important role in antioxidants may play a protective role in ICH.

## Therapeutic potential of nuclear factor erythroid-2-related factor 2 and nuclear factor erythroid-2-related factor 2 pathway activators in the treatment of intracerebral hemorrhage

Nuclear factor erythroid-2-related factor 2 lies at the center of a complex regulatory network, its activity can be controlled at the transcriptional and post-transcriptional levels by regulating gene levels, post-translational modifications, protein stability, subcellular localization, and others. Many activators of Nrf2 and the Nrf2 pathway can promote the expression of Nrf2 and promote the translocation of Nrf2 from the cytoplasm to the nucleus, thereby transcriptionally activating the expression of downstream antioxidant and anti-inflammatory genes. Here, we summarize the effects of several Nrf2 and Nrf2 pathway activators from *in vivo* experimental ICH models (Table 1).

**TABLE 1:** The activators of Nrf2 and Nrf2 pathway in experimental ICH models.

	Compounds	Model	Targets	Efficacy	References
Nrf2 activators	Sulforaphane	Autologous blood-induced rat/mouse ICH model	Keap1	CAT ↑, SOD ↑, NQO1 ↑, GST ↑, 3′-NT ↓, 4-HNE ↓, neurologic deficits ↓	Zhao et al., 2007
	(−)-Epicatechin	Collagenase-induced mouse ICH model	Keap1	SOD1 ↑, NQO1 ↑, neurologic deficits ↓	Chang et al., 2014
	Isoliquiritigenin	Collagenase IV-induced rat ICH model	Keap1	GSH ↑, SOD ↑, CAT ↑, ROS ↓, GSSG ↓, 3-NT ↓, 8-OHdG ↓, neurologic deficits ↓	Zeng et al., 2017
	Luteolin	Autologous blood-induced rat ICH model	Keap1	HO-1 ↑, NQO1 ↑, neurologic deficits ↓	Tan et al., 2019
	Dimethyl fumarate	Collagenase- or autologous blood-induced rat/mouse ICH model	Keap1, p-Nrf2	HO-1 ↑, NQO1 ↑, CAT ↑, brain edema ↓, neurologic deficits ↓	Iniaghe et al., 2015; Zhao et al., 2015
	Curcumin	Autologous blood-induced rat ICH model	Keap1	GSH ↑, HO-1 ↑, NQO1 ↑, Gpx4 ↑, ROS ↓, MDA ↓, intracranial hematoma ↓	Dinkova-Kostova et al., 2001
	RS9	Autologous blood-induced mouse ICH model	Keap1, p-Nrf2	HO-1 ↑, SOD-1 ↑, brain edema ↓, neurologic deficits ↓	Sugiyama et al., 2018
Activators of Nrf2 pathway	Phillyrin	Collagenase IV-induced mouse ICH model	Nrf2 signaling pathway	HO-1 ↑, NQO1 ↑, SOD-1 ↑	Guo et al., 2021
	Polydatin	Autologous blood-induced rat ICH model	Nrf2 signaling pathway	HO-1 ↑, NQO1 ↑, SOD ↑, GSH ↑, brain edema ↓, neurologic deficits ↓	Zhao et al., 2020
	Crocin	Autologous blood-induced mouse ICH model	Nrf2 signaling pathway	SOD ↑, GSH-px ↑, MDA ↓, GPX4 ↑, FTH1 ↑, SLC7A11 ↑, brain edema ↓, neurologic deficits ↓	Wang et al., 2022
	Ghrelin	Autologous blood-induced mouse ICH model	Nrf2 signaling pathway	NQO1 ↑, GCLC ↑, SOD1 ↑, MDA ↓, brain edema ↓, neurologic deficits ↓	Cheng et al., 2020
	Albumin	Autologous blood-induced rat ICH model	p-Nrf2, Nrf2 signaling pathway	HO-1 ↑, long-term neurological deficit ↓	Deng et al., 2021

### Nuclear factor erythroid-2-related factor 2 activators

Most Nrf2 activators act to alter Keap1 primarily by binding to multiple cysteines in the Kelch domain of Keap1, thereby affecting the Nrf2/Keap1 complex and preventing Nrf2 ubiquitination and degradation. After Nrf2 dissociates from the Nrf2/Keap1 complex, it is translocated from the cytoplasm to the nucleus, where Nrf2 transcriptionally activates the expression of antioxidant, anti-inflammatory and detoxification genes (McMahon et al., 2003; Hong et al., 2005).

Sulforaphane is a sulfur-rich dietary phytochemical that displays antioxidant and anti-inflammatory properties in various neurological disorders (Uddin et al., 2020). Sulforaphane, an Nrf2 activator, induces the nuclear translocation and activation of Nrf2, thereby activating the Nrf2 pathway after ICH. Activation of Nrf2 with sulforaphane increases antioxidative and detoxifying enzymes and reduces oxidative damage and inflammation in brain areas affected by an intraparenchymal hematoma. These properties suggest that sulforaphane may be a promising therapeutic for ICH (Zhao et al., 2007).

(−)-Epicatechin is a natural brain-penetrating product that is abundant in green tea and cocoa; it modulates redox/oxidative stress by activating the Nrf2 pathway. Previous studies have shown that oral consumption of (−)-epicatechin promotes vascular function, cognition, and attenuates ischemic brain injury in animals and humans (Schroeter et al., 2006; Shah et al., 2010). Recent studies demonstrated that (−)-epicatechin significantly reduces lesion volume and ameliorates neurologic deficits in a mouse model of ICH. Oral treatment with (−)-epicatechin alleviated ICH-induced brain oxidative injury and increased nuclear Nrf2 accumulation and phase II enzyme (SOD1 and NQO1) expression in the brains of ICH mice. As such, it exerts a protective role against hemorrhagic brain injury through Nrf2 activation (Chang et al., 2014).

Isoliquiritigenin is a natural flavonoid isolated from licorice root; it has antioxidant, anti-inflammatory, and neuroprotective functions. Isoliquiritigenin has been found to significantly alleviate neurological deficits, BBB disruption, brain edema, and neuronal degeneration by activating Nrf2 after ICH. Isoliquiritigenin can promote the expression and nuclear translocation of Nrf2, and suppress the activation of NF-κB and NLRP3 inflammasome pathways 24 h after ICH, thereby exerting antioxidant and anti-inflammatory effects in a collagenase IV-induced rat ICH model (Zeng et al., 2017).

Luteolin is a natural flavonoid found in a variety of plants and shows various pharmacological activities. Luteolin alleviates brain edema and ameliorates neurobehavioral dysfunction and memory loss *in vivo*. Studies have shown that luteolin increased the expression and nuclear translocation of Nrf2 to activate the expression of downstream antioxidant proteins, such as HO-1, and reduced NADPH: NQO1 24 h after ICH. In addition to acting as an Nrf2 activator, luteolin also enhanced p62-dependent cell autophagy and inhibited the ubiquitination and degradation of Nrf2 (Tan et al., 2019).

Dimethyl fumarate is a fumaric acid ester previously shown to be a neuroprotective drug and tested in clinical trials for patients with relapsing-remitting multiple sclerosis. It has been determined that dimethyl fumarate promotes Nrf2 activation and stabilization through direct modification of Keap1 at cysteine residue 151 (Linker et al., 2011). Recently studies have shown that dimethyl fumarate can alleviate neurological deficits and brain edema after ICH. Dimethyl fumarate treatment increased the expression of Nrf2 and the downstream antioxidant proteins HO-1, NQO1, and CAT after ICH. Further, dimethyl fumarate also promoted an anti-inflammatory response by reducing the secretion of IL-1β and iNOS, and increasing the expression of the anti-inflammatory cytokine IL-10 (Zhao et al., 2015). Interestingly, dimethyl fumarate increased the expression of CK2 in the cytoplasm while promoting Nrf2 phosphorylation in the nucleus, indicating that dimethyl fumarate simultaneously modified Keap1 and Nrf2, thereby promoting the translocation of Nrf2 into the nucleus (Iniaghe et al., 2015).

Curcumin was originally isolated from *Curcuma longa* L., a naturally occurring polyphenol with a low molecular weight. Studies have shown that curcumin reacts with the sulfhydryl group in the Keap1 protein to break the hydrogen bond in Keap1, thereby reducing the affinity of Keap1 and Nrf2. Therefore, curcumin can act as an Nrf2 activator, enabling Nrf2 to enter the nucleus to play a transcriptional regulatory role in promoting downstream gene transcription (Dinkova-Kostova et al., 2001). Recently, curcumin was found to reduce the water content of perihematomal brain tissue and effectively promote the clearance of intracranial hematoma after ICH by activating Nrf2 (Duan et al., 2022b). Curcumin treatment significantly increased intracranial Nrf2 expression, further decreased ROS and MDA levels in brain tissue, and increased levels of GSH, HO-1, NQO1, Gpx4, and SOD. This indicates that curcumin promotes the translocation of Nrf2 into the nucleus by suppressing Keap1 activity, thereby transcriptionally activating the downstream antioxidant pathway.

RS9 is a novel Nrf2 activator that is biotransformed from bardoxolone methyl (BARD). RS9 acts as an Nrf2 activator with a mechanism of action similar to BARD in interacting with Keap1 to prevent Nrf2 ubiquitination and accelerating its translocation into the nucleus. RS9 was shown to decrease brain edema, improve neurological deficits, decrease neuronal damage area, and increase the expression of HO-1 and SOD-1 in an ICH mouse model (Sugiyama et al., 2018). The protective effects of RS9 against ICH damage occur by promoting the activation of Nrf2 to upregulate the expression of downstream antioxidant genes. Moreover, RS9 was shown to upregulate the expression of phosphorylated Akt (p-Akt), which is strongly related to the Nrf2 signal. It has been reported that increasing the expression of p-Akt suppresses Nrf2 degradation and accelerates the expression of antioxidant genes (Xin et al., 2018). Thus, RS9 exerts neuroprotective effects in an Akt-Nrf2-antioxidant pathway dependent manner.

### Agents of active nuclear factor erythroid-2-related factor 2 pathway

There are some compounds for which direct evidence of Keap1 activation to affect the Nrf2/Keap1 complex is lacking; these compounds promote the expression of Nrf2 and the translocation of Nrf2 from the cytoplasm to the nucleus by modifying protein stability or its subcellular localization.

Phillyrin is an active ingredient extracted from the traditional Chinese herb *Forsythia* (Thunb.) Vahl (Oleaceae). Previous studies showed that phillyrin protects against oxidative stress damage by activating the Nrf2 signaling pathway. Recently, it was found that phillyrin is neuroprotective in ICH *via* its activation of the Nrf2 signaling pathway (Guo et al., 2021). Phillyrin significantly reduced lesion volume, improved the white and gray matter injury around the lesion, decreased apoptosis and oxidative stress, increased the expression of Nrf2, HO-1, NQO1, and SOD-1 in *in vitro* and *in vivo* models, and protected neurons from hemin stimulation by promoting Nrf2 nuclear translocation (Guo et al., 2021).

Polydatin is a glycoside of resveratrol isolated from the rhizome of *Polygonum cuspidatum*. It has been determined to activate the Nrf2/ARE pathway and shows neuroprotective effects (Huang et al., 2018; Lv et al., 2019). The antioxidant mechanism of polydatin may stem from its activation of PKC and Sirt1, the upstream regulators of Nrf2, thereby activating Nrf2 and its downstream antioxidant pathways (Ma et al., 2016; Qiao et al., 2016). Previous studies demonstrated that promotion of the Nrf2/ARE signaling pathway protects against ischemic stroke injury *in vivo* by mitigating oxidative stress, apoptosis, and inflammatory response after spinal cord ischemia/reperfusion injury (Zhan et al., 2021). Recently, polydatin was found to upregulate antioxidant target gene enzymes and downregulate detoxification enzymes by activating Nrf2 to protect against ICH brain injury. Polydatin treatment reduced the levels of NO and MDA, and increased the expression of Nrf2, HO-1, and NQO1 in rat brains after ICH. This indicates that polydatin reduces brain tissue edema and protects against nerve cell injury in ICH by activating the Nrf2 pathway (Zhao et al., 2020).

Crocin is a carotenoid isolated from *Crocus sativus* L. It possesses potential anti-inflammatory and antioxidative functions in a variety of diseases of the central nervous and cardiovascular systems. Several studies have shown that crocin reduces oxidative stress, inflammation, and apoptosis by promoting the Nrf2/HO-1 pathway (Khodir et al., 2019; Liang et al., 2020). Recently, crocin was reported to improve the outcome of ICH. Crocin treatment reduced brain edema and alleviated neurological deficits after ICH, increasing SOD and GSH and reducing MDA. Furthermore, crocin treatment also increased the expression of GPX4, FTH1 and SLC7A11, regulating Nrf2 and reducing ferroptosis in ICH. Thus, crocin can alleviate ICH-induced oxidative stress and neuronal ferroptosis by facilitating Nrf2 nuclear translocation (Wang et al., 2022).

Ghrelin is a novel endogenous brain-gut peptide consisting of 28 amino acids that acts as an endogenous ligand for the growth hormone secretagogue receptor. Studies have shown that ghrelin is transported across the BBB and provides neuroprotection in ischemic stroke (Cheyuo et al., 2011). Recently, ghrelin has been reported to attenuate neurobehavioral deficits, brain edema, hematoma volume, and perihematomal cell death after ICH. Ghrelin treatment significantly upregulated the expression and nuclear accumulation of Nrf2, accompanied by increased expression of Nrf2 downstream target antioxidant genes (Cheng et al., 2020). Furthermore, Ghrelin can also suppress the neuroinflammatory response by inhibiting NLRP3 inflammasome activation and the resultant downstream neuroinflammatory cascade. Therefore, ghrelin may exert its neuroprotective effects by enhancing the Nrf2/ARE antioxidant signaling pathway and inhibiting NLRP3 inflammasome activation.

Albumin is a single non-glycosylated polypeptide chain of 585 amino acids, which has been regarded as a potent antioxidant with free radical scavenging properties. Previous studies have demonstrated that treatment with human serum albumin imparts neuroprotective effects on focal and global cerebral ischemia, subarachnoid hemorrhage, and ICH (Belayev et al., 2001, 2005; Xie et al., 2017). Recently, studies showed albumin reduces oxidative stress and neuronal apoptosis in ICH rats by activating the ERK/Nrf2/HO-1 pathway (Deng et al., 2021). Albumin treatment ameliorated long-term neurological deficits while increasing ERK1/2 phosphorylation and Nrf2 and HO-1 expression in a rat ICH model. The ERK inhibitor U-0126 reversed the albumin-induced ERK1/2 phosphorylation and the increased Nrf2 and HO-1 expression. This indicates that albumin exerts its protective effects on ICH in part by activating the ERK/Nrf2/HO-1 signaling pathway.

## Conclusion

**FIGURE 1:**
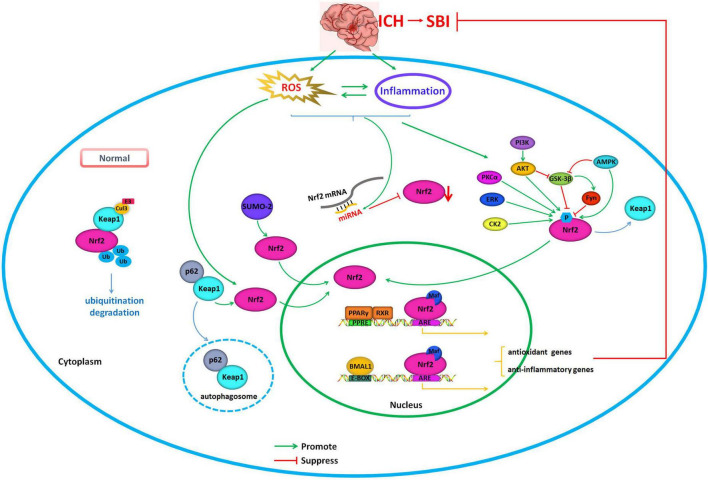
Regulation of Nrf2 as the potential therapeutic target in intracerebral hemorrhage. Under normal physiological conditions, Nrf2 interacts with Keap1 and is anchored in the cytoplasm. Keap1 serves as a substrate for Cul3/Rbx1 E3 ubiquitin, which can promote the ubiquitination of Nrf2 resulting in its rapid degradation from the proteasome, which serves to maintain Nrf2 levels. During conditions that produce oxidative stress, including excessive production of ROS, Nrf2, and Keap1 disconnect, and Nrf2 translocates into the nucleus. There, Nrf2 can coordinate anti-oxidative stress and anti-inflammatory processes by means of AREs that are located in target gene regulatory regions. The activity of Nrf2 can be controlled by regulating gene expression, protein stability, subcellular localization, and synergistic effect with other transcription factors. Green arrows indicate promotion of Nrf2 translocation into the nucleus and activation. Red arrows indicate suppressing of Nrf2 translocation into the nucleus and inhibition of its activity.

In addition to neuroinflammation, oxidative stress is a critical factor that leads to SBI after ICH. Oxidative stress is involved in a number of essential aspects of the physiological and pathological responses that take place during ICH. Nrf2 can coordinate anti-oxidative stress and anti-inflammatory processes by means of AREs that are located in target gene regulatory regions, which plays an important role in reducing brain damage after ICH. Thus, the regulation of Nrf2 is a potential therapeutic target in ICH. Nrf2 is at the center of a complex regulatory network. Its activity can be controlled at the transcriptional and post-transcriptional levels by regulating gene expression levels, protein stability, subcellular localization, and synergistic effects with other transcription factors (Figure 1). Future studies should explore the neuroprotective effects of the Nrf2 pathway in response to ICH and ICH-induced SBI to identify new drugs and drug targets that can be exploited to effectively treat ICH.

Even though various Nrf2 activators were shown to be effective in the treatment of ICH in *in vitro* and *in vivo* models, and a few have been approved for the clinical treatment of other diseases, more effective drugs need to be identified that can attenuate the cascade of damage in perihematoma tissues and that lack systemic side effects. Systematic preclinical and clinical safety are required to confirm the efficacy and safety of Nrf2 activators in the treatment of ICH and provide a pathway for the clinical use of Nrf2 activators in ICH.

In summary, activation of Nrf2 might produce antioxidant, anti-inflammatory, and neuron-protection effects, which could potentially be a focus for developing future treatments and prevention of ICH.

## Author contributions

YZ conceived and wrote the manuscript. YZ, WY, YL, WC, MW, and LZ reviewed and edited the manuscript. All authors have read and approved the manuscript.

## Abbreviations

3 ′ -UTR: 3 ′ untranslated region
Akt: protein kinase B
AMPK: AMP-activated protein kinase
ARE: antioxidant response element
BARD: bardoxolone methyl
BMAL1: brain and muscle Arnt-like protein 1
bZIP: basic leucine zipper
CAT: catalase
CK2: casein kinase 2
CNC: cap-n-collar
Cul3: Cullin3
ERK1/2: extracellular signal-regulated kinases 1/2
GSK-3 β: glycogen synthase kinase 3 β
GST: glutathione S-transferase
HO-1: heme oxygenase-1
ICH: intracerebral hemorrhage
IL-10: interleukin-10
NADPH: nicotinamide adenine dinucleotide phosphate
NF- κ B: nuclear factor- κ B
NQO1: quinine oxidoreductase 1
Nrf2: nuclear factor erythroid-2-related factor 2
PDZ: pioglitazone
PHE: perihematomal edema
PI3K: phosphatidylinositol-4,5-bisphosphate 3-kinase
PKC: protein kinase C
PPAR γ: peroxisome proliferator-activated receptor γ
PPRE: peroxisome proliferator response element
RBC: red blood cell
RNS: reactive nitrogen species
ROS: reactive oxygen species
RXR: retinoid X receptor
SBI: secondary brain injury
SOD: superoxide dismutase
SUMO: small ubiquitin-related modifier
TNF- α: tumor necrosis factor α
XAR: xenobiotic-activated receptor.
